# Supplementary material for: Engineering multifunctional rhizosphere probiotics using consortia of Bacillus amyloliquefaciens transposon insertion mutants
Source: eLife. 2023 Sep 14;12:e90726. doi: 10.7554/eLife.90726 (PMC10519709; doi:10.7554/eLife.90726)
Supplement: Supplementary file 2. — (a) Comparison of in vitro traits between mutants belonging to three clusters using unpaired two-samples Wilcoxon test. Significant effects (p<0.05) are highlighted in bold. (b) Comparison of root colonization and plant protection between mutants belonging to three clusters using unpaired two-samples Wilcoxon test. Significant effects (p<0.05) are highlighted in bold and dpi denotes for days post-pathogen inoculation. (c) Comparison of trait values of eight mutants used for the assembly of consortia richness gradient relative to the wild-type strain based on Student’s t-test. Significant differences are shown in bold and arrows show increase (upward) and decrease (downward) in trait values. (d) p-Values for comparing the biomass production of each mutant strain on its own or other strains’ supernatant compared to fresh 50% LB based on Student’s t-test. Significant differences are shown in bold and arrows show facilitative (upward) and antagonistic (downward) interactions between the mutants. The magnitude of these interactions is shown in Figure 3—figure supplement 1 as a heatmap. (e) Comparison of mutant identity effects on consortia root colonization and plant protection based on the absence and presence of each mutant in consortia. Significant effects (p<0.05) are highlighted in bold based on unpaired two-samples Wilcoxon test. (f) Comparison of the mutant identity effects and consortia richness on root colonization and plant protection. Richness was fitted sequentially after mutant identity effects (presence or absence in consortia). Both response variables were treated as continuous variables and Bacillus abundance data was log-transformed before the analysis. Significant effects (p<0.05) are highlighted in bold. (g) Bacterial strains and plasmid used in this study. (h) Primers used in this study. [file elife-90726-supp2.docx]

**Supplementary file 2a. Comparison of *in vitro* traits between mutants belonging to three clusters using unpaired two-samples Wilcoxon test.** Significant effects (p < 0.05) are highlighted in bold.

| **Mutant trait measured *in vitro*** | **Comparison** | **W** | **P** |
| --- | --- | --- | --- |
| Swarming motility | Cluster1 (n=265) VS Cluster2 (n=112) | **11125** | **<0.001** |
|  | Cluster1 (n=265) VS Cluster3 (n=103) | **26874** | **<0.001** |
|  | Cluster2 (n=112) VS Cluster3 (n=103) | **11517** | **<0.001** |
| Biomass production | Cluster1 (n=265) VS Cluster2 (n=112) | **10869** | **<0.001** |
|  | Cluster1(n=265) VS Cluster3 (n=103) | **7754** | **<0.001** |
|  | Cluster2 (n=112) VS Cluster3 (n=103) | 4931 | 0.0664 |
| Biofilm formation | Cluster1 (n=265) VS Cluster2 (n=112) | **23061** | **<0.001** |
|  | Cluster1 (n=265) VS Cluster3 (n=103) | **18268** | **<0.001** |
|  | Cluster2 (n=112) VS Cluster3 (n=103) | **4238** | **<0.001** |
| Pathogen suppression | Cluster1 (n=265) VS Cluster2 (n=112) | **28464** | **<0.001** |
|  | Cluster1 (n=265) VS Cluster3 (n=103) | **27253** | **<0.001** |
|  | Cluster2 (n=112) VS Cluster3 (n=103) | **10300** | **<0.001** |

**Supplementary file 2b. Comparison of root colonization and plant protection between mutants belonging to three clusters using unpaired two-samples Wilcoxon test.** Significant effects (p < 0.05) are highlighted in bold and dpi denotes for days post-pathogen inoculation.

| **Mutant trait measured *in vivo*** | **Comparison** | **30 dpi** | |
| --- | --- | --- | --- |
|  |  | **W** | **P** |
| Root colonization  (*B. amyloliquefaciens* T-5 abundance; log_10_ cells g^-1^ rhizosphere soil) | Cluster1 (n=27) VS Cluster2 (n=11) | **233.5** | **0.0065** |
|  | Cluster1 (n=27) VS Cluster3 (n=10) | **193.5** | **0.0472** |
|  | Cluster2 (n=11) VS Cluster3 (n=10) | 42 | 0.3867 |
| Plant protection  (Disease incidence; % of wilted plants) | Cluster1 (n=27) VS Cluster2 (n=11) | **85.5** | **0.0442** |
|  | Cluster1 (n=27) VS Cluster3 (n=10) | **24.5** | **<0.001** |
|  | Cluster2 (n=11) VS Cluster3 (n=10) | 31.5 | 0.1052 |

**Supplementary file 2c. Comparison of trait values of eight mutants used for the assembly of consortia richness gradient relative to the wild-type strain based on students’ t-test.** Significant differences are shown in bold and arrows show increase (upwards) and decrease (downwards) in trait values.

| **Mutant strain** | **P-values and changes in trait value relative to wild-type strain** | | | |
| --- | --- | --- | --- | --- |
|  | Swarming  motility | Biomass  production | Biofilm  formation | Pathogen  suppression |
| M38 | 0.3486 | **0.0262↓** | **0.0298↓** | **0.0408↑** |
| M54 | 0.1836 | **0.0035↓** | **0.0193↑** | 0.5513 |
| M59 | **< 0.001↓** | **< 0.001↑** | **0.0084↓** | **< 0.001↓** |
| M78 | **< 0.001↓** | **< 0.001↓** | 0.3608 | **0.0128↑** |
| M108 | **0.0016↑** | **0.0181↓** | **0.0176↓** | 0.7541 |
| M109 | **0.0147↓** | **0.0014↑** | **0.0069↓** | 0.8217 |
| M124 | **< 0.001↑** | **0.0125↓** | **0.0033↓** | **0.0206↓** |
| M143 | **< 0.001↓** | **< 0.001↓** | **< 0.001↑** | 0.7541 |

| **Growing** | **Supernatant source** | | | | | | | | |
| --- | --- | --- | --- | --- | --- | --- | --- | --- | --- |
| **mutant strain** |  |  |  |  |  |  |  |  |  |
|  | **M38** | **M54** | **M59** | **M78** | **M108** | **M109** | **M124** | **M143** | **WT** |
| **M38** | **0.0489↓** | **0.0202** | **0.0092↓** | 0.7062 | **0.0085↓** | **0.0023↓** | 0.6659 | **0.0441↓** | 0.0521 |
| **M54** | **0.0098↓** | 0.1632 | **0.0051↓** | **0.0299↓** | **0.0251↓** | **0.0125↓** | **0.0060↓** | **0.0136↓** | **0.0028↓** |
| **M59** | 0.7637 | **0.0028↓** | 0.0739 | **<0.001↓** | 0.0589 | **0.0152↓** | **<0.001↓** | **0.0021↓** | **0.0014↓** |
| **M78** | **0.0011↓** | **0.0166↓** | **0.0065↓** | 0.9239 | **0.0080↓** | **0.0032↓** | **0.0255↓** | **0.0065↓** | **0.0026↓** |
| **M108** | 0.1515 | 0.0917 | **0.0113↓** | 0.6877 | **0.0271↓** | **0.0213↓** | 0.8150 | 0.2301 | **0.0154↓** |
| **M109** | 0.1709 | 0.1517 | 0.6975 | 0.4325 | 0.1562 | 0.0522 | 0.2466 | 0.9747 | **0.0363↓** |
| **M124** | **0.0227↓** | **0.0015↓** | 0.5282 | 0.4584 | 0.1536 | **0.0233↓** | **<0.001↑** | **0.0023↑** | 0.0710 |
| **M143** | 0.7815 | 0.3179 | 0.0630 | 0.4702 | 0.2432 | 0.2227 | **0.0246↓** | 0.0606 | 0.4774 |
| **WT** | **0.0148↓** | **0.0010↓** | **0.0012↓** | **<0.001↓** | **0.0446↓** | **<0.001↓** | **0.0013↓** | **0.0016↓** | **0.0045↓** |

**Supplementary file 2d. P-values for comparing the biomass production of each mutant strain on its own or other strains’ supernatant compared to fresh 50% LB based on student’s t-test.** Significant differences are shown in bold and arrows show facilitative (upwards) and antagonistic (downwards) interactions between the mutants. The magnitude of these interactions is shown in Figure 3–figure supplement 1 as a heatmap.


**Supplementary file 2e. Comparison of mutant identity effects on consortia root colonization and plant protection based on the absence and presence of each mutant in consortia.** Significant effects (p < 0.05) are highlighted in bold based on unpaired two-samples Wilcoxon test.

|  |  |  | **Root colonization**  **(*Bacillus* abundance)** | |  | **Plant protection**  **(Disease incidence)** | |
| --- | --- | --- | --- | --- | --- | --- | --- |
| **Mutant**  **strain**  **identity** | **Phenotype** | **Comparison** | **W** | **P** |  | **W** | **P** |
| M38 | Pathogen suppression+ | Included (n=12) VS Excluded (n=25) | 197 | 0.1314 |  | 116 | 0.2815 |
| M54 | Biofilm formation+ | Included (n=12) VS Excluded (n=25) | **266** | **0.0143** |  | 128 | 0.4909 |
| M59 | Biomass production+ | Included (n=12) VS Excluded (n=25) | 192.5 | 0.1729 |  | **78** | **0.0188** |
| M78 | Pathogen suppression+ | Included (n=12) VS Excluded (n=25) | 139 | 0.7333 |  | 113 | 0.2401 |
| M108 | Swarming motility+ | Included (n=12) VS Excluded (n=25) | 154 | 0.9096 |  | 120 | 0.3435 |
| M109 | Biomass production+ | Included (n=12) VS Excluded (n=25) | 166.5 | 0.6037 |  | 105 | 0.1507 |
| M124 | Swarming motility+ | Included (n=12) VS Excluded (n=25) | 177 | 0.3899 |  | 97 | 0.0887 |
| M143 | Biofilm production+ | Included (n=12) VS Excluded (n=25) | 155 | 0.8839 |  | **78** | **0.0188** |

**Supplementary file 2f. Comparison of the mutant identity effects and consortia richness on root colonization and plant protection.** Richness was ﬁtted sequentially after mutant identity effects (presence or absence in consortia). Both response variables were treated as continuous variables and *Bacillus* abundance data was log-transformed before the analysis. Significant effects (p < 0.05) are highlighted in bold.

|  |  |  | **Root colonization (*Bacillus* abundance)** | |  | **Plant protection (Disease incidence)** | |
| --- | --- | --- | --- | --- | --- | --- | --- |
| **Mutant**  **strain**  **identity** | **Phenotype** | ***Df*** | ***F*** | ***P*** |  | ***F*** | ***P*** |
| M38 | Pathogen suppression+ | 1 | **4604.70** | **<0.001** |  | **652.86** | **<0.001** |
| M54 | Biofilm formation+ | 1 | **3413.15** | **<0.001** |  | **476.77** | **<0.001** |
| M59 | Biomass production+ | 1 | **2665.22** | **<0.001** |  | **433.88** | **<0.001** |
| M78 | Pathogen suppression+ | 1 | **1719.05** | **<0.001** |  | **269.91** | **<0.001** |
| M108 | Swarming motility+ | 1 | **2021.15** | **<0.001** |  | **272.12** | **<0.001** |
| M109 | Biomass production + | 1 | **1515.47** | **<0.001** |  | **215.33** | **<0.001** |
| M124 | Swarming motility+ | 1 | **1431.96** | **<0.001** |  | **185.12** | **<0.001** |
| M143 | Biofilm formation + | 1 | **1035.52** | **<0.001** |  | **126.80** | **<0.001** |
| Richness |  |  | **9.07** | **0.0048** |  | **22.24** | **<0.001** |
| Linear term |  | 1 |  |  |  |  |  |
| *Error* |  | 29 |  |  |  |  |  |

**Supplementary file 2g. Bacterial strains and plasmid used in this study.**

| **Strain or plasmid** | **Characteristics** | **Source** |
| --- | --- | --- |
| *Ralstonia solanacearum* QL-Rs1115 | Causative agent of Bacterial wilt | Z. Wei *et al*. 2011 (76) |
| *Bacillus amyloliquefaciens* T-5 | Rhizosphere isolate capable of suppressing the growth of *Ralstonia solanacearum* | S. Y. Tan *et al*. 2013 (45), X. F. Wang *et al*. 2017 (94) |
| pMarA plasmid | promoter σA, kmR ApR EmR pUC19 carrying TnYLB-1 transposon, mariner-Himar1 transposase | Y. Le Breton *et al*. 2006 (47) |

**Supplementary file 2h. Primers used in this study.**

| **Primer** | **Sequence (5’ to 3’ end)** | **Source or reference** |
| --- | --- | --- |
| oIPCR1 | GCTTGTAAATTCTATCATAATTG | Y. Le Breton *et al*. 2006 (47) |
| oIPCR2 | AGGGAATCATTTGAAGGTTGG |  |
| oIPCR3 | GCATTTAATACTAGCGACGCC |  |
